# Modifying the Catalyst Layer Using Polyvinyl Alcohol for the Performance Improvement of Proton Exchange Membrane Fuel Cells under Low Humidity Operations

**DOI:** 10.3390/polym12091865

**Published:** 2020-08-19

**Authors:** Prathak Jienkulsawad, Yong-Song Chen, Amornchai Arpornwichanop

**Affiliations:** 1 Center of Excellence in Process and Energy Systems Engineering, Department of Chemical Engineering, Faculty of Engineering, Chulalongkorn University, Bangkok 10330, Thailand; prathak.j@gmail.com (P.J.); amornchai.a@chula.ac.th (A.A.); 2 Department of Mechanical Engineering and Advanced Institute of Manufacturing with High-tech Innovations, National Chung Cheng University, Chiayi County 62102, Taiwan

**Keywords:** proton exchange membrane fuel cell, humidifier, membrane electrode assembly, polyvinyl alcohol

## Abstract

A proton exchange membrane fuel cell (PEMFC) system for the application of unmanned aerial vehicles is equipped without humidifiers and the cathode channels of the stack are open to the environment due to limited weight available for power sources. As a result, the PEMFC is operated under low humidity conditions, causing membrane dehydration, low performance, and degradation. To keep the generated water within the fuel cell to humidify the membrane, in this study, polyvinyl alcohol (PVA) is employed in the fabrication of membrane electrode assemblies (MEAs). The effect of PVA content, either sprayed on the gas diffusion layer (GDL) or mixed in the catalyst layer (CL), on the MEA performance is compared under various humidity conditions. The results show that MEA performance is increased with the addition of PVA either on the GDL or in the CL, especially for non-humidified anode conditions. The result suggested that 0.03% PVA in the anode CL and 0.1% PVA on the GDL can improve the MEA performance by approximately 30%, under conditions of a non-humidified anode and a room-temperature-humidified cathode. However, MEAs with PVA in the anode CL show better durability than those with PVA on the GDL according to measurement with electrochemical impedance spectroscopy.

## 1. Introduction

Proton exchange membrane fuel cells (PEMFCs) have gained much attention for transportation applications, especially in unmanned aerial vehicles (UAVs) [1], due to their low operating temperature (<100 °C), high power density, and high energy density. Presently, as the commercial PEMFC uses a Nafion membrane which needs to be hydrated for facilitating proton transfer, a humidification for the supplied air is recommended to avoid membrane drying [2]. However, carrying a humidifier is a challenge for small UAVs (e.g., multirotors), which have space and weight limits. In this case, the membrane might have to be hydrated by moisture in the air, which is relatively low at the temperature of the PEMFC, or by the water generated during the electrochemical reaction. Many researchers have attempted to modify the membrane electrode assembly (MEA) of the PEMFC and enhance its performance under a low humidity condition or self-humidification [3].

A way to improve MEAs for low humidity operation involves adding some additives into either the membrane or the catalyst layer (CL). The additives should have the ability to hold water, which could be hydrophilic materials, such as SiO_2_ [4], TiO_2_ [5] or polyvinyl alcohol (PVA) [6], have hydrophilic functional groups such as triazole [7] and vinyl phosphonic acid (VPA) [8], or be a metal-organic framework (MOF) [9]. In addition to mixing the additive into the CL or membrane composite, use of dual CLs has been investigated [10]. Moreover, functionalizing a carbon electrode can also enhance the hydrophilic property of the electrode [11,12]. However, the process of CL modification seems to be simpler compared to that of membrane modification. SiO_2_ is mostly used as an additive in the CL in many research investigations due to the high ability of silica to increase the water uptake. Su et al. [13] successfully added SiO_2_ into the CL on a Nafion 212 membrane by using the organic colloid method for catalyst ink preparation and the illumination method for spraying and forming a CL. The results of the analysis showed excellent bonding among silica, Pt, and C. No significant difference in the performance of the PEMFC using the anode with or without SiO_2_ under external humidification condition was observed, indicating that SiO_2_ did not affect the electrochemical reaction. However, a long-term operation (20 h) of PEMFCs with 3 wt.% SiO_2_ in the anode showed a drop in performance of 22% with a very stable power of 438 mW cm^−2^. Han et al. [14] fabricated MEAs with 6 wt.% SiO_2_ by using the hot press method to make contact between CLs and the membrane. It was found that the performance of the PEMFC was lower than that reported by Su et al. [13], even though the higher SiO_2_ content (6 wt.%) was used. Hence, the method of MEA fabrication has a significant impact on cell performance. MEAs with the catalyst coated membrane (CCM) show better performance than those with the catalyst coated GDL. These results were validated by the study of Leimin et al. [15]. The CCM under irradiation (CSMUI) method showed better contact between the catalyst and membrane, resulting in small cell resistances (total ohmic resistance and charge transfer resistance), compared to the catalyst coated GDL method.

PVA has been widely used in membrane modification because of its high water absorption capability and electrical resistance [6]. However, PVA has low proton conductivity and thus phosphorylated PVA, which has a crosslinking structure, was often used, as it has relatively high proton conductivity and still has water retention ability [16]. El-Toony et al. [17] cast a membrane of phosphorylated polyvinyl alcohol (p-PVA)/poly hydroxybutyrate (PHB) for PEMFCs using the gamma irradiation method for making p-PVA. The membrane was cast in polystyrene petri dishes (solution casting method) and the CLs were assembled with the membrane by the hot-pressed method. The cast membrane showed superior performance compared to Nafion 212 under a relative humidity of 100% in both short- and long-term tests. The maximum power density achieved was about 639 mW cm^−2^. However, the PEMFC was not tested under a low relative humidity condition in their study. Attaran et al. [18] cast PVA/polyvinyl pyrrolidone (PVP)/BaZrO_3_ by using the solution casting method. Glutaraldehyde solution, the crosslinking agent, was used to prepare the PVA. The ceramic material BaZrO_3_, with perovskite structure, was added to further improve proton conductivity. The painting method was applied to coat catalyst on carbon paper, and then the hot-pressing method was applied to assemble the MEA. Although the proton conductivity was improved, the cell performance degraded (28.98 mW cm^−2^). In addition to adding PVA into a membrane composite, Liang et al. [19] added PVA into the anode CL. They used the illuminated spraying method on the Nafion 212, with a controlled active area of 5 cm^2^. The MEA with PVA 5 wt.% was found to be optimal to achieve the maximum power density of 623.3 mW cm^−2^, with relatively low ohmic resistance and the lowest charge transfer of the MEAs under low humidification (RH 34%) and pressurized condition (20 psi). PVA in CLs shows a remarkable high performance, similar to PVA in the membrane (with the appropriate preparation method), and it is comparative to other additives.

Using PVA as a water absorbent in the anode CL has been investigated at the pressured condition of 20 psi; however, the effect of PVA on the MEA performance under ambient environment needs to be further studied for UAV applications. In this study, performance of MEAs with various PAV concentrations in the anode CL (named the PA method) is evaluated under various cell temperatures and humidifier temperatures. Moreover, coating a thin layer of PVA on the anode GDL (named the PG method) as a water reservoir for hydrating the membrane is proposed in this study. The durability of these MEAs with different configurations is also studied using electrochemical impedance spectroscopy.

## 2. Experimental

### 2.1. MEA Fabrication

The MEA consists of a CCM sandwiched between two GDLs (GDL260, CeTech, Taichung City, Taiwan). Catalyst ink was a mixture of 46.8 wt% Pt on carbon (TEC10E50E, Tanaka, Japan), polyvinyl alcohol (72000 BioChemica, AppliChem GmbH, Darmstadt, Germany), Nafion dispersion (D520, Chemours Fluoroproducts), ethanol (95% ethanol, TTL Taiwan), and deionized water. The catalyst ink was mixed in a planetary centrifugal mixer (Thinky mixer, ARE-310, Tokyo, Japan) for 30 min, followed by an ultrasonic bath (Delta D80, Taipei, Taiwan) for 10 min, and again mixed in the ARE-310 for 30 min. The catalyst ink was sprayed on both sides of the membrane (Nafion NR211, Dupont, Wilmington, DE, USA) with an active area of 26.01 cm^2^ by using an ultrasonic spraying system (Benchtop BT, USI, USA). The solvent was evaporated during spraying process at 100 °C by using a hot plate (PC-400D, Corning, New York, NY, USA).

**Figure 1:**
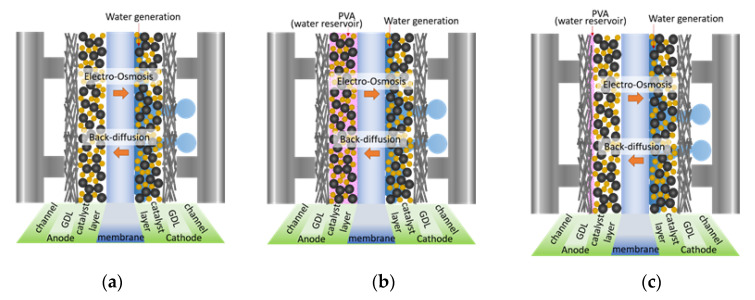
Schematic of a single cell with different electrode configurations: (**a**) no polyvinyl alcohol (PVA) case, (**b**) PVA in the anode catalyst layer (CL), and (**c**) PVA on the gas diffusion layers (GDLs).

The Nafion to carbon ratio in the catalyst ink was predetermined as 1:1 though a preliminary study to study the effect of the PVA location on the performance of the PEMFC under low humidity operation. Pt loadings in the anode and cathode CLs were controlled to be 0.1 mg cm^−2^ and 0.3 mg cm^−2^, respectively. PVA (0.1 wt.% solution) was prepared by dissolving PVA monomer in DI water at 90 °C. PVA in the CL was 0.01, 0.03, 0.07 and 0.1 wet weight%, using the PA method, based on a preliminary trial. Cathode catalyst ink was prepared by the same method, except no PVA was added. Regarding the PG method, PVA solution was sprayed on the GDL, while the anode catalyst without PVA was coated on the membrane. The amount of PVA on the GDL was controlled as done for the anode CL (the PA method), to study the effect of the different preparation methods. The MEA was assembled by sandwiching the CCM between two GDLs without a hot-pressing step and a Teflon gasket with a thickness of 0.225 mm was used to prevent gas leakage. The schematic of MEAs of a PEMFC, for this study, is shown in Figure 1.

### 2.2. Performance Test

A fuel cell test station (HS-330s, Hephas Energy Corporation, Hsinchu, Taiwan) with an electronic load (PLZ164WA, Kikusui, Yokohama, Japan) was used for both the activation process and performance tests. Before a performance test, each MEA was activated for 12 h at the fuel cell temperature of 60 °C, anode and cathode humidifier temperatures were set at 50 °C, H_2_ with a stoichiometric ratio of 1.2 was used, the minimum flow rate was 100 mL min^−1^, and the air was prepared with a stoichiometric ratio of 3 and a minimum flow rate of 200 mL min^−1^. During the activation process, the load was periodically changed with a minimum voltage of 0.42 V.

In the performance test, H_2_ and air settings during the test were the same as during the activation. However, the fuel cell was tested in both non-humidified anode (dry anode) and humidified anode (wet anode) modes. The test conditions are shown in Table 1. During the test, voltage was measured while the current density was increased from 0 to 1.2 A cm^−2^ with a step of 0.1 A cm^−2^.

## 3. Results and Discussion

### 3.1. Effect of PVA on MEA Performance

**Figure 2:**
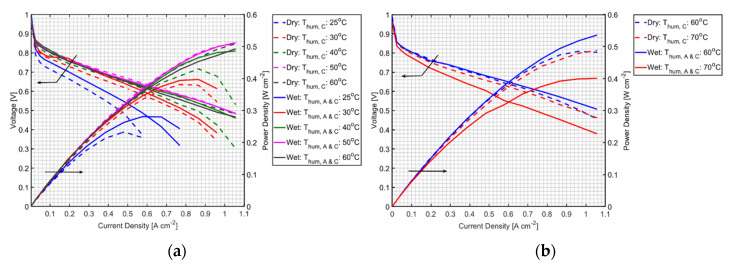
Performances of membrane electrode assemblies (MEAs) without PVA under non-humidified and humidified anode conditions, at a fuel cell temperature of (**a**) 60 °C and (**b**) 70 °C.

Figure 2 shows the performances of MEAs without PVA operated at various humidifier temperatures. It can be seen that when both humidifier temperatures were lower than cell temperature, humidifying anode hydrogen can significantly improve the cell performance (solid lines performance is better than that of the dashed lines). However, when the humidifier temperature approached cell temperature, whether the anode was humidified or not, there was no significant effect on the cell performance, as shown in Figure 2a. Similar results can be observed at the cell temperature of 70 °C. When the cathode humidifier temperature was 60 °C, both MEAs under dry and humidified hydrogen conditions showed similar performance. However, at fully humidified conditions, in which the both the anode and cathode humidifier temperatures were the same as the cell temperature, the MEA showed a notable performance drop due to water flooding within the cell. Water existing in the CL is in both liquid and gas form. The liquid water comes with the saturated gas feed and from water absorbed by Nafion in the CL [20]. In addition to the flooding, the performance is lost by lower O_2_ concentration in the saturated gas [21].

**Figure 3:**
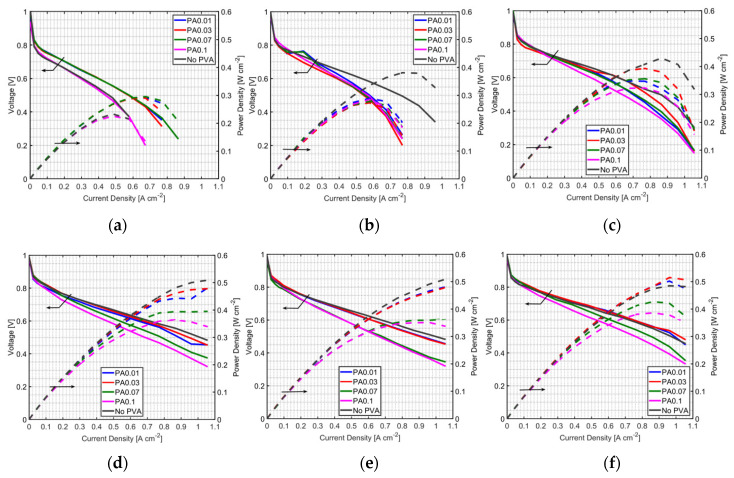

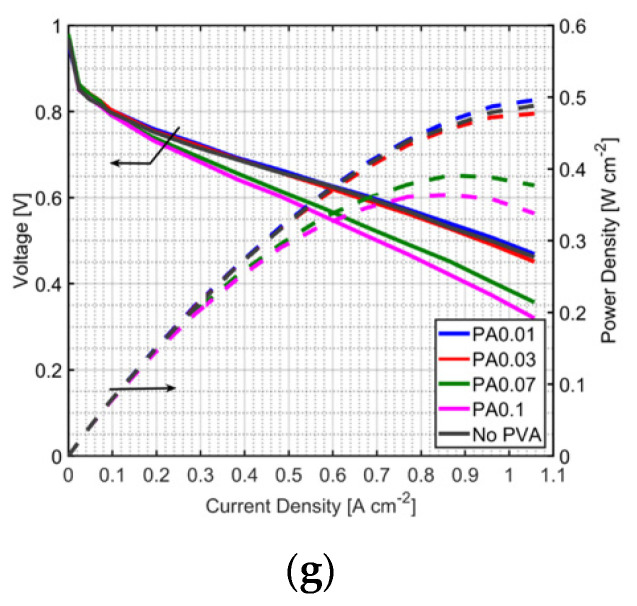
Performances of MEAs with the PA method at a fuel cell temperature of 60 °C, and with a non-humidified anode and cathode humidifier temperature of (**a**) 25 °C, (**b**) 30 °C, (**c**) 40°C, (**d**) 50 °C and (**e**) 60 °C; and at a fuel cell temperature of 70 °C, with a non-humidified anode, and a cathode humidifier temperature of (**f**) 60 °C and (**g**) 70 °C.

**Figure 4:**
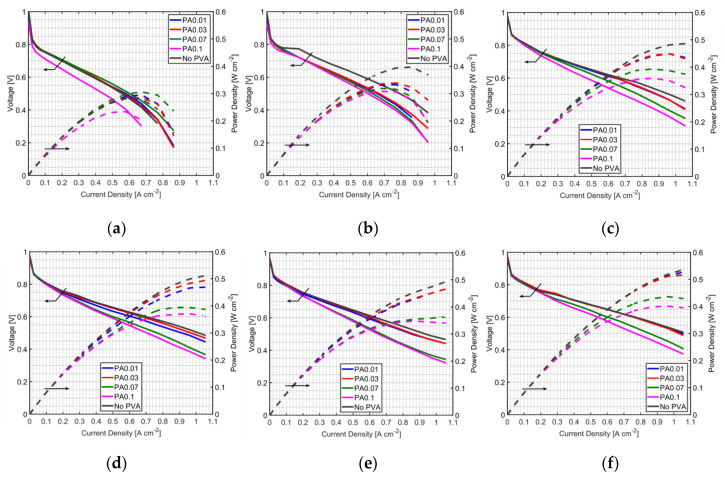

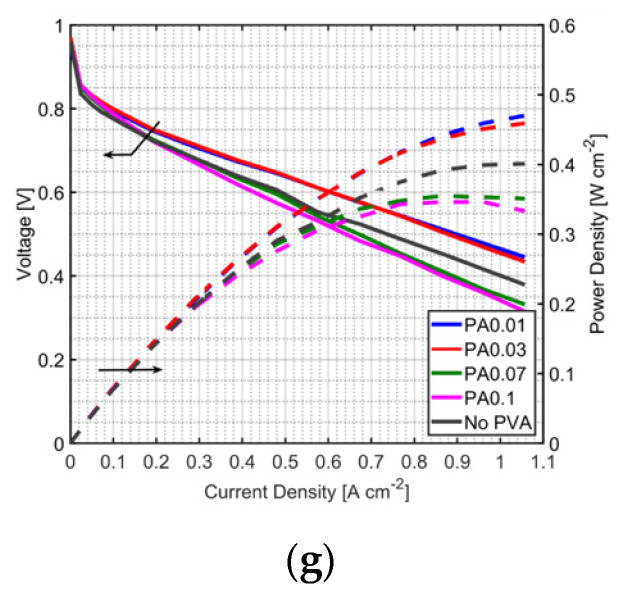
Performances of MEAs prepared with the PA method at a fuel cell temperature of 60 °C and the humidifier temperature of the anode and the cathode at (**a**) 25 °C, (**b**) 30 °C, (**c**) 40 °C, (**d**) 50 °C and (**e**) 60 °C; and at a fuel cell temperature of 70 °C and the humidifier temperature of the anode and the cathode at (**f**) 60 °C and (**g**) 70 °C.

For PVA-modified MEAs, the PEMFC performance depends on the PVA content. Table 2 shows the PVA content in MEAs in this study. Performances of MEA with PVA in the anode CL (PA) under non-humidified anode operation are shown in Figure 3a–e for a fuel cell temperature of 60 °C and in Figure 3f,g for a fuel cell temperature of 70 °C. Performances of PEMFC prepared by the PA method under humidified anode operation is shown in Figure 4a–e and Figure 4f,g for a fuel cell temperature of 70 °C and 60 °C, respectively. It is found that the PA method improved PEMFC performance only at a very low humidifier temperature, 25 °C, in both non-humidified and humidified anode cases. This result indicates that PVA is an effective additive to retain water, even at low humidity conditions. However, the addition of PVA showed a negative effect on the fuel cell performance when used at high humidify conditions (higher than the humidifier temperature of 25 °C); the performance of PEMFCs using the PA method was lower than that without PVA-MEA at other cathode humidifier temperatures (Figure 3b–e and Figure 4b–e). It may be caused by PVA properties as a proton exchanger and electrical resistance material; adding PVA can enhance these properties of MEA. In addition, PVA might cover the catalyst active area, and the captured water may block fuel to react with the catalyst, leading to an increase in the concentration loss. Therefore, the use of the PA method could be a good way to improve the PEMFC performance when it is operated at very low humidity conditions and with a dry anode.

For a fuel cell temperature of 70 °C, low PVA loading in the anode (PA0.01 and PA0.03) showed no different performance, compared to the case of PVA-free MEA, except at a humidified anode and humidifier temperature of 70 °C. This may be because the amount of PVA caused a water balance between water transport by electro-osmosis and water diffused by back-diffusion at this operating condition. Higher operating temperature reduced ohmic loss of the fuel cell as the slope of the V-I curve at a fuel cell temperature of 70 °C and a humidifier temperature of 60 °C is less steep than that at a fuel cell temperature of 60 °C and a humidifier temperature of 60 °C. Thus, the addition of a smaller amount of PVA in the anode under this operating condition results in performance enhancement.

### 3.2. Effect of PVA Location in the MEA

**Figure 5:**
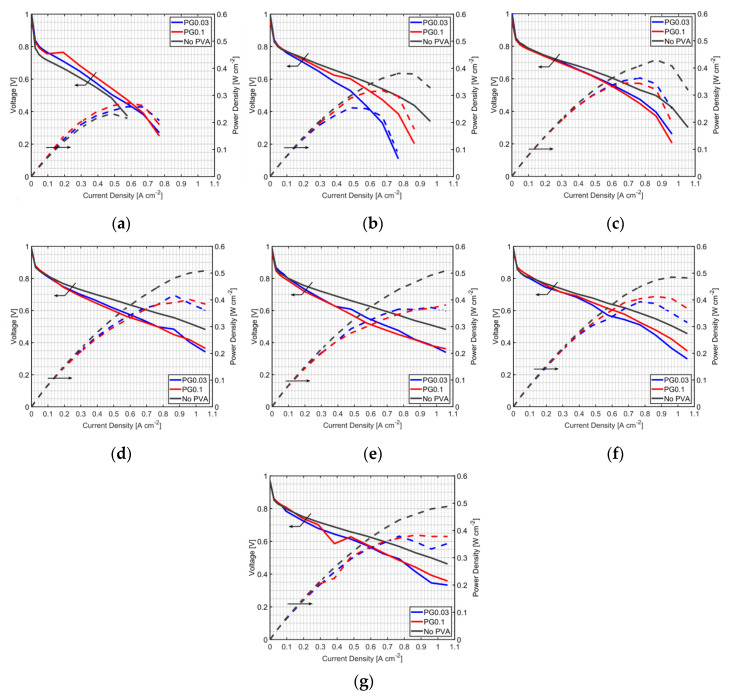
MEA with the PG method at a fuel cell temperature of 60 °C, with a non-humidified anode and a cathode humidifier temperature of (**a**) 25 °C, (**b**) 30 °C, (**c**) 40 °C, (**d**) 50 °C and (**e**) 60 °C; and a fuel cell temperature of 70 °C, a non-humidified anode and a cathode humidifier temperature of (**f**) 60 °C and (**g**) 70 °C.

**Figure 6:**
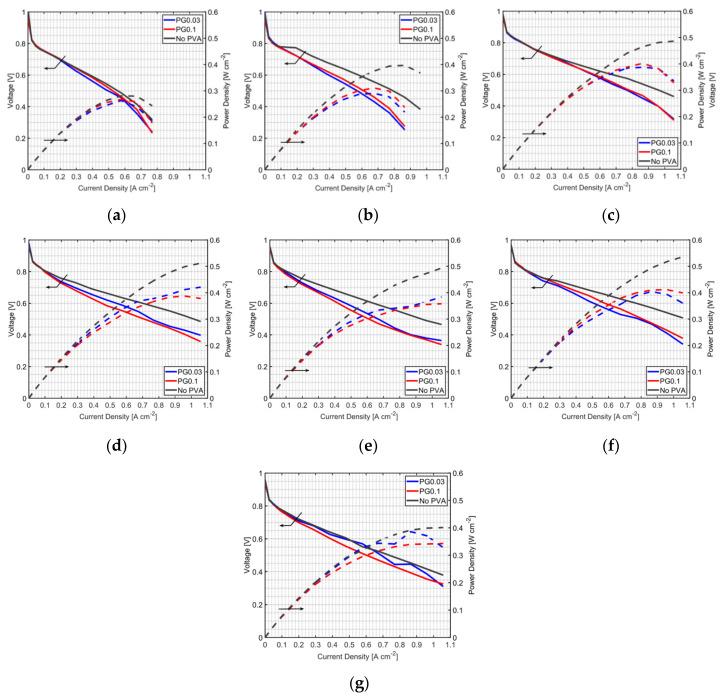
MEA with the PG method at a fuel cell temperature of 60 °C, and an anode and cathode humidifier temperature of (**a**) 25 °C, (**b**) 30 °C, (**c**) 40 °C, (**d**) 50 °C and (**e**) 60 °C; and a fuel cell temperature of 70 °C, and an anode and cathode humidifier temperature of (**f**) 60 °C and (**g**) 70 °C.

Because PVA causes the increase of resistance in MEAs when it is added into the anode, a new method of applying PVA, which was modified from a double layer-based technique, was proposed in this study. Instead of adding PVA into the anode catalyst, it was sprayed on the GDL as a layer between the GDL and anode CL. Due to an ultra-thin anode CL, the PVA layer could be wet by water from the back-diffusion through the membrane. However, it also could be a film between the GDL and anode CL, and it would be difficult for gas to pass through. The schematic of PVA on GDLs is shown in Figure 1c; the PVA layer is just a water reservoir, not a reaction layer. In order to compare with the PA method, PVA loading on GDLs was controlled by the number of spraying cycles, so as to have the amount of PVA close to that in the PA method. In this study, one cycle of PVA spraying on the GDL contained PVA of 0.18366 μg cm^−2^. Table 2 shows the PVA loading of PG cases compared with PA cases. However, it is difficult to spray a PG case having the amount of PVA close to the PA0.01 case due to the very low content of PVA. Although the PG method does not affect the reaction at the anode CL, it can improve the PEMFC performance under low humidified conditions and dry anode conditions (Figure 5 and Figure 6). PVA on GDLs can hold water from the water back-diffusion as the PEMFC performance in non-humidified anode cases (Figure 5) was changed with the moisture content in the cathode side. However, too much moisture content in the cathode side might also cause difficulty in gas transport through GDL with PVA. At low moisture content with a humidifier temperature of 25 °C, gas can go through the PVA layer, and performance is enhanced with PVA at this condition. It is found that the PG0.1 case gives better performance compared to PG0.03 due to high PVA loading at this condition; more water was adsorbed with high PVA content on the GDL and high proton exchange can be achieved at this condition. Improved PEMFC using the PG method with low PVA loading may have less resistance, but higher wettability seems to be more important to enhance the PEMFC performance. All humidified anode PG cases (Figure 6), however, show a negative effect on the performance improvement, whereas PA cases can slightly improve fuel cell performance at a humidifier temperature of 25 °C. The PVA layer is assumed to be a barrier for fuel transportation when the anode is humidified. Moisture in the anode can make the PVA swollen and results in less porosity at the surface of the GDL. Therefore, the PG method is not recommended for humidified anode application.

**Figure 7:**
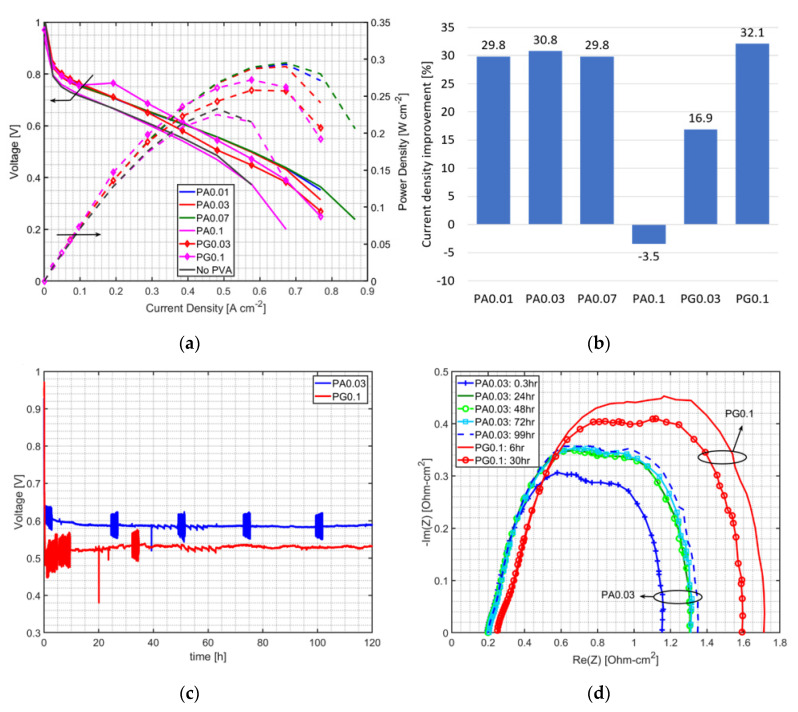
(**a**) Comparison of PEMFCs using PVA-MEA prepared by the PA and PG methods at a non-humidified anode and cathode humidifier temperature of 25 °C, (**b**) percent improvement of PVA-MEA from MEA without PVA at the non-humidified anode, with a cathode humidifier temperature of 25 °C and a voltage of 0.6 V, (**c**) the durability test and (**d**) the impedance curve of PA0.03 and PG0.1 at a constant current density of 0.4 A cm^−2^, with a non-humidified anode and a cathode humidifier temperature of 25 °C.

Figure 7a shows that with the use of a small amount of PVA into MEAs, both the PVA in the anode (PA) and PVA on GDL (PG) methods can effectively enhance the PEMFC performance when it is operated at low humidity and non-humidified anode conditions. Note that PA0.1 gives a PEMFC performance similar to using MEA without PVA, whereas PG0.1 can improve the PEMFC. It indicates that this amount of PVA using the PA method can hinder the catalyst active area, even though the membrane is wet. However, this similar amount of PVA using the PG method did not affect the catalyst active area, and thus it enhanced the performance. In comparison with PA0.03 and PG0.03, PA0.03 provided better performance if the current density was higher than 0.3 A cm^−2^. It might be because the PVA content in the anode was able to keep the membrane hydrated, and dominated the effect of PVA on the catalyst surface area. Thus, PVA location in the MEA plays an important role in cell improvement. PVA was preferred to be a bit distant from the membrane when higher PVA loading was used, as shown in the PA0.1-PG0.1 cases. For lower PVA loadings like the PA0.03-PG0.03 cases, PVA was instead placed inside the anode CL close to the membrane. The PEMFC improvement by PVA at a cell voltage of 0.6 V, a non-humidified anode, and a cathode humidifier temperature of 25 °C is shown in Figure 7b. Adding a small amount of PVA into the MEA can improve the PEMFC current density by around 30% at selected operating conditions.

### 3.3. Durability Test

For a durability test, both methods are examined by comparing the voltage variation of those MEAs at the constant current density of 0.4 A cm^−2^, which is the result of short-term test conditions to achieve the target voltage of 0.6 V (Figure 7a), and constant feed flow rates for 120 h. However, no durability test of the MEA without PVA is presented in Figure 7c,d because the performance of the MEA without PVA showed a lower performance than the PA0.03 and PG0.1 cases. According to Figure 7a, the voltage of the MEA without PVA can start from 0.55V when the current density is at 0.4 A cm^−2^, and a quick performance drop will occur under dry operating conditions; therefore, no long term test data can be provided for the MEA without PVA with the target voltage of 0.6 V and a given constant current density of 0.4 A cm^−2^. PA0.03 and PG0.1 are selected as representative of the PA and PG methods, respectively, due to highest performance improvement in each method. Although PG0.1 showed a slightly higher performance than PA0.03 (Figure 7b), greater voltage drop (about 13.3%) is found in the PG case in a long term test (Figure 7c); whereas voltage drops of about 3.4% were found in the PA case. Impedance curves of the PA and PG methods (Figure 7d) were measured at the current density of 0.4 A cm^−2^ by electrochemical impedance spectroscopy with frequency sweeping from 10 mHz to 10 kHz. It shows that the PA method has smaller resistance, both membrane resistance (ohmic loss) and polarization resistance (activation losses and mass transport losses), than the PG method. Higher resistance in the PG case might be because higher PVA loading was used in the PG case, and fuel had more difficulty passing through a swollen PVA layer when water was accumulated with time; this is observed from the polarization resistance increase with time (Figure 7d). The periodic fluctuation in voltage was due to the measurement of electrochemical impedance, in which the frequency was changed. In comparison with PVA-MEA using a similar fabrication technique (spraying method), the achieved maximum power density proposed by Liang et al. [19] is higher than the PVA-MEA prepared by both the PA and PG methods. The lower achievement could be due to differences in operating conditions and material compositions. However, the proposed methods in this study can be used in the lower humidity condition (RH of 15.9% and a non-humidified anode). Roh et al. [12] tested the oxygen-group functionalized carbon supported dual catalyst layer of PEMFC under RH of 26%. The results were superior to the proposed method in this study. However, it is more complicated and costly for PEMFC fabrication.

## 4. Conclusions

This study is aimed at improving the performance of PEMFCs operated at an ambient environment with low humidity, using PVA as an additive. An ultrasonic spraying method was employed to coat a CL on a Nafion 211 membrane. PVA was applied to the MEA by two methods: PVA in the anode and PVA on GDLs (a newly proposed method). PVA was added in the anode during the catalyst ink preparation procedure for PVA in the anode (PA) case. For PVA on the GDL (PG), PVA was sprayed on the anode GDL with the same coating machine. Although the use of PVA can increase the wettability of the MEA’s PEMFC, low PVA content was required to minimize the cell resistance caused by the PVA. Both PA and PG methods can enhance the PEMFC performance only at very low humidity condition, especially under non-humidified anode condition. The current density of the PVA-MEA prepared by the PA and PG methods could be improved by approximately 30%, at the operating voltage of 0.6 V, and non-humidified anode and cathode humidifier temperature of 25 °C. However, the durability test revealed that the PA method is more durable than the PG method, due to a smaller voltage drop.

## Figures and Tables

**Table 1:** Temperatures of the fuel cell and anode/cathode humidifiers.

Case		Fuel Cell Temperature (°C)	Humidifier Temperature (°C) (Relative Humidity RH %)	
			Anode	Cathode
Wet	1.	60	25 (15.9)	25 (15.9)
	2.	60	30 (21.3)	30 (21.3)
	3.	60	40 (37.0)	40 (37.0)
	4.	60	50 (61.9)	50 (61.9)
	5.	60	60 (100)	60 (100)
	6.	70	60 (63.9)	60 (63.9)
	7.	70	70 (100)	70 (100)
Dry	8.	60	non-humidified	25 (15.9)
	9.	60	non-humidified	30 (21.3)
	10.	60	non-humidified	40 (37.0)
	11.	60	non-humidified	50 (61.9)
	12.	60	non-humidified	60 (100)
	13.	70	non-humidified	25 (10.2)
	14.	70	non-humidified	70 (100)

**Table 2:** PVA-MEA denotation.

Case	Description	PVA Loading (μg cm^−2^)
PA0.01	PVA 0.01 wt.% in anode CL	0.03274
PA0.03	PVA 0.03 wt.% in anode CL	0.09823
PA0.07	PVA 0.07 wt.% in anode CL	0.22931
PA0.1	PVA 0.1 wt.% in anode CL	0.32762
PG0.03	PVA 0.5 cycles * on GDL which has PVA loading close to PA0.03 case	0.09183
PG0.1	PVA 1.75 cycles * on GDL which has PVA loading close to PA0.1 case	0.32140

*: 1 cycle of spraying PVA on GDL contains PVA of 0.18366 μg cm^−2^.
